# Distribution of low-molecular lipophilic extractives beneath the surface of air- and kiln-dried Scots pine sapwood boards

**DOI:** 10.1371/journal.pone.0204212

**Published:** 2018-10-10

**Authors:** Olena Myronycheva, Olov Karlsson, Margot Sehlstedt-Persson, Micael Öhman, Dick Sandberg

**Affiliations:** Division of Wood Science and Engineering, Luleå University of Technology, Skellefteå, Sweden; Bangor University, UNITED KINGDOM

## Abstract

During industrial wood drying, extractives migrate towards the wood surfaces and make the material more susceptible to photo/biodegradation. The present work provides information about the distribution, quantity and nature of lipophilic substances beneath the surface in air- and kiln-dried Scots pine (*Pinus sylvestris* L.) sapwood boards. Samples were taken from knot-free sapwood surfaces and the composition of lipophilic extractives, phenols and low-molecular fatty/resin acids layers at different nominal depths below the surface was studied gravimetrically, by UV-spectrometry and by gas chromatography-mass spectrometry (GC-MS). The concentration of total extractives was significantly higher in kiln-dried than in air-dried samples and was higher close to the surface than in the layers beneath. The scatter in the values for the lipophilic extractives was high in both drying types, being highest for linoleic acid and slightly lower for palmitic, oleic and stearic acids. The amount of fatty acids was low in kiln-dried boards, probably due to a stronger degradation due to the high temperature employed. The most abundant resin acid was dehydroabietic acid followed by pimaric, isopimaric, and abietic acids in both drying types. It is concluded that during kiln-drying a migration front is created at a depth of 0.25 mm with a thickness of about 0.5 mm.

## Introduction

Wood is dried industrially to remove water to achieve a more durable and dimensionally stable light-weight material. Drying and especially thermal modification influence the presence and structure of the constituents of the wood material, and subsequently the characteristics and service-life of the final wood products. During drying, complex changes in the properties of wood occur, initiated by heat and mass flows towards the wood surfaces and by degradation of wood constituents [1]. One mechanism is a migration of non-structural wood-cell compounds such as polar and non-polar extractives. Contamination of both hydrophilic and hydrophobic components beneath and at the wood surface by the migration of extractives during drying reduces the adhesion of surface coatings [2,3], affects the speed of moisture absorption [4], leads to an uneven distribution of preservatives during impregnation [5], and as a consequence may reduce the durability of the wood product.

Scots pine (*Pinus sylvestris* L.) is one of the major raw materials for the forest products industries in the Nordic countries. Scots pine is naturally highly enriched in extractives, which influence the modification of sawn timber and the properties of the final product. The migration of hydrophilic extractive fractions to the sapwood surface during drying and thermal modification has been widely described [6–9]. The movement of liphophilic extractives during drying decreases the permeability and can lead to failure in impregnation [5,10]. Besides, the hydrophobic fractions that remain from nutrient reserves and natural defensive systems against pathogens in the living tree can be used as natural protection of wood products against damage by environmental influences.

The main wood constituents are cellulose, hemicelluloses, lignin, and to a minor part water-soluble and insoluble extracellular and low-molecular-weight compounds called “extractives” [11,12]. The extractives are located in various tree compartments: parenchyma and epithelial cells, resin canals, middle lamellae, intercellular spaces and the cell walls of tracheids, libriform fibers and vessels [13]. The main compounds in softwoods are resin acids, other terpenoids, fats, fatty acids, steryl esters, sterols, phenolic substances, glycosides, sugars, starch, and proteins [14] with seasonal and geographical variations in distribution pattern [15–17]. The content of extractives has been reported to be higher in knots than in stem wood, with noted variation within the tree and between trees and a certain decrease in amount along the radial direction of the tree [18].

The resins in Scots pine are primary lipid-soluble blends of volatile and non-volatile secondary compounds of terpenoid origin, produced in subcellular regions of epithelial cells and then transported and stored in the resin duct [19]. The volatile fractions of the mono C_10_ and sesquiterpene C_15_ resins give fluidity to resins and plasticise the more viscous C_20_ diterpenes. Scots pine possesses the high level of constitutive resins in an interconnected system of resin ducts located in the stem wood with long-lived epithelial cells from the initial state with dominance in heartwood. The non-volatile resin fraction of Scots pine is usually characterized by diterpene resin acids of the abietane and pimarane structural types [20]. The predominant phenolic compounds are flavonoids, stilbenes and lignans. Their main function is to prevent oxidation of biomolecules by abiotic and biotic substances attacking the trees [21]. The pinosylvin stilbenes are the predominant phenolic compounds in Scots pine heartwood and also exist in sapwood [22]. Triglycerides, such as esters of glycerol and three fatty acids C_16_ and C_18_ with varying degrees of unsaturation and sterol/triterpenoid esters in Scots pine, are stored in sapwood with higher concentrations of free fatty acids then in the heartwood, probably related to degradation of triacylglycerols during heartwood formation [23]. Unsaturated fatty acids such as oleic, linoleic, linolenic and eicosatrienoic, and the saturated palmitic and stearic fatty acids, are the most abundant components of the triacylglycerol fraction [23,24].

Green wood retains metabolic activity after harvesting and processing, and this initiates a natural anatomical response to injuries that trees sustain while exposed to pathogenic attack or mechanical damage. It results in the release of constitutive defense agents and activates complex changes in non-structural elements of Scots pine wood [25]. The moisture flux during the drying process re-locates low-molecular sugars [7,26] beneath the sawn timber surface with the formation of a so-called drying shell and kiln brown stain [6] that can be explained by the hypothesis of an evaporative front (about 10 cell rows) of capillary water beneath the surface [27].

Despite the large amount of research in recent decades into the extractives in Scots pine, only limited and their fragmented information can be found regarding the distributionof hydrophobic/lipophilic extractives and migration towards the surface during drying or thermal treatment. Moreover, the accumulation and differences in chemical composition of extractives in relation to the contribution of compound migration remains unclear. The present work has sought to contribute information about the distribution, quantity and nature of some of the hydrophobic substances in air- and kiln-dried Scots pine sapwood.

## Material and methods

### Specimen preparation and drying procedure

A total of 10 green sideboards from 10 different Scots pine (*Pinus sylvetris* L.) trees with cross-sectional dimensions of 220 x 25 mm, and a length of about 4.5 metres were taken directly after sawing from a sawmill in Norrbotten County in Sweden during the last week of September 2015. The trees were felled, bucked to logs and transported to the sawmill during September. Each board was cut into four pieces with a length of 0.96 m and the moisture content (green state) was determined according to EN 13183–1 [28]. The boards consisted only of sapwood.

No permits were required for the study, which complied with all relevant regulations. The study did not involve endangered or protected species.

The boards were divided into two groups for air-drying and kiln-drying as follows: ten randomly selected boards were single stacked and dried indoors on stickers at a temperature of 20°C (RH about 10%) and used as reference for extractives analysis as air-dried samples. The average moisture content after drying for 30 days was 4.6%.

**Fig 1:**
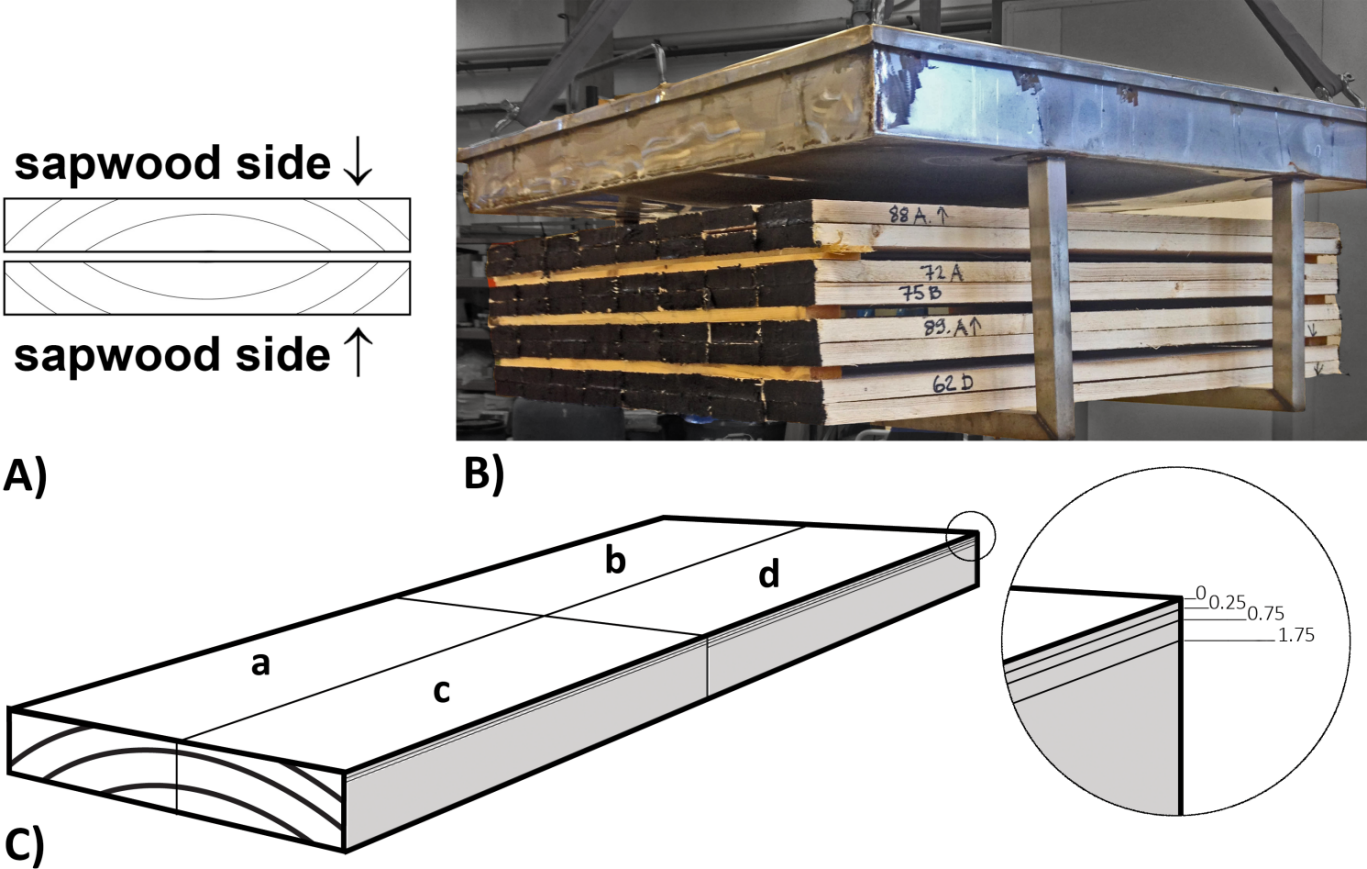
Drying and board preparation scheme. (A) double-stacking of boards with the sapwood side of each board facing outwards in each pair, (B) stacking of boards in the laboratory kiln, and (C) planing depth scheme for specimens: (a) unplaned, (b) 0–0.25 mm, (c) 0.25–0.75 mm, and (d) 0.75–1.75 mm planing depth.

**Fig 2:**
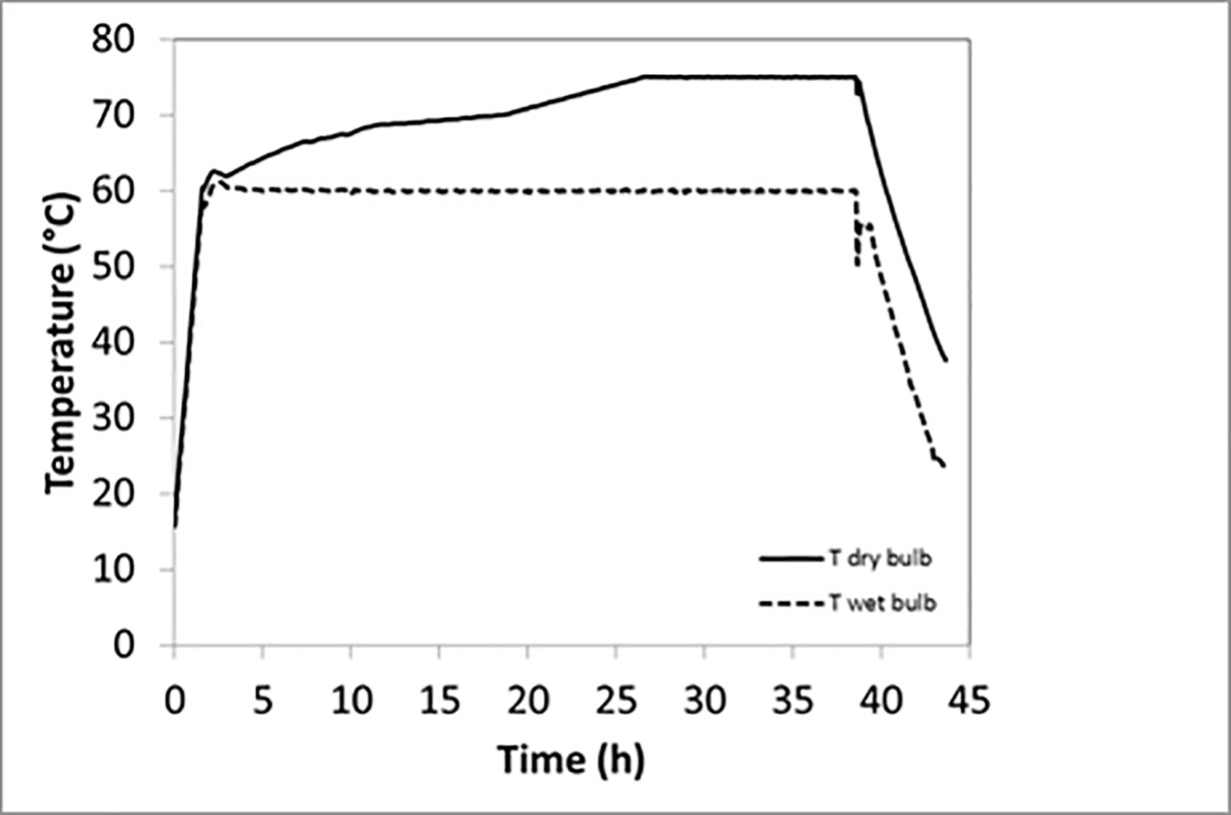
Drying schedule showing dry- and wet-bulb temperatures. The total drying time was 44 hours including a 1.7 hours heating phase, and a 5 hour cooling phase.

Another 64 boards were kiln-dried in a small-scale laboratory kiln with air circulation. All the boards were double-stacked with the sapwood sides of each pair turned outwards in order to get a high flow of moisture from the inner part of the boards to the sapwood surface that could enrich the surfaces with as much extractives as possible during drying (Fig 1). The drying schedule used is shown in Fig 2 and the moisture content (MC) is shown in Table 1.

**Table 1:** Moisture content of the kiln-dried bards.

Moisture content	Mean±SD (SE), %
Before drying	116**±**19.6 (6.2)
After drying	14**±**3.8 (1.2)

The kiln-drying was performed without a conditioning phase in order to prevent influence on distribution of extractives after drying.

### Extraction of extractives and analysis

All the air-dried boards and 10 randomly selected kiln-dried boards were cut to a length of 250 mm and a width of 100 mm, and then split into four specimens (Fig 1C). These specimens were used for the determination of the distribution of lipophilic extractives. The sapwood sides of three of four specimens were planed three times to depths of 0.25, 0.75 and 1.75 mm from the original un-planed surface. 3.5 g of the shavings from each planing were milled in a Fritch planetary mill and 0.5 g was used for moisture content determination, 1.0 g was used for the extraction of lipophilic compounds, and the remainder was left untreated and stored at a temperature of -20°C.

The extraction was done using acetone (VWR Chemicals: 20165.323, 75 ml) in a mini-Soxhlet extractor according to the SCAN-CM 49:03 standard method [29]. The total lipophilic extractives content was given in mg g^-1^ of dry mass after evaporation of the acetone and drying of the extracted solution.

Fatty acids and resin acids in the acetone extract were analysed using GC-MS after trimethylsilylation. The silylation with 100 μl of BSTFA and 50 μl of TMSCl was performed in an oven at 70°C for 20 minutes according to Sjöström [30] with 1-methylnaphthalene dissolved in pyridine as internal standard [31].

A SUPELCO SLB-5 MS capillary column (L × I.D. 30 m × 0.25 mm, d_f_ 0.25 μm) was used starting at a temperature of 100°C (1 min initial) and finishing at 270°C (2 min final) at a rate 10°C/min with an entire analysis cycle of 25 minutes. The injector initial temperature was 270°C with interface temperature 290°C and fan temperature was 50°C, control mode was split. The scan group started from 40 m/z and end 500 m/z with scan speed 1000.

The resin acid and fatty acids were identified by NIST Mass Spectral Library [32] and a quantitative estimation was made by comparing the peak areas with that for the internal standard. Retention time (RT) was: hexanoic acid (RT ~ 5.6 min.), heptanal (RT ~ 6.2 min.), glycerol (RT ~ 8.6 min.), internal standard (RT ~ 9.4 min.), palmitic acid (RT ~ 17.9 min.), oleic acid (RT ~ 19.6 min.), linoleic acid (RT ~ 19.4 min.), stearic acid (RT ~ 19.7 min.), pimaric acid (RT ~ 20.4 min.), isopimaric acid (RT ~ 20.8 min.), dehydroabietic acid (RT ~ 21.2 min.), abietic acid (RT ~ 21.5 min.).

The total phenolics content was determined by Folin-Ciocalteu (FC) assay according to a procedure described by Julkunen-Tiitto [33]. The absorption was measured after 40 minutes at 735 nm against that of a mixture containing all the reagents except for the sample. Tannic acid (VWR Chemicals; 83510.260) was used as a standard and the results are expressed in mg (TAE) g^-1^ dry mass of wood. The protocol was deposited in protocols.io, where it can be assigned via link http://dx.doi.org/10.17504/protocols.io.phqdj5w.

### Data analysis

The complete data matrix with 60 observations and 9 variables as measurement parameters was based on 10 specimens in each drying method with 3 planing depths Table 2.

**Table 2:** Sample groups with 60 observations.

Planing depth (mm)	No. of replicates	
	Air-dried	Kiln-dried
0–0.25	10	10
0.25–0.75	10	10
0.75–1.75	10	10

The relative mean difference (Δ mean in %) was determined by subtracting the mean of the kiln-dried variable from that of the air-dried variable and dividing by the air-dried mean, the coefficient of variation (CV in %) was used to describe the dispersion around the mean. The variables measured were: extractives, phenolics, glycerol, palmitic oleic-, linoleic- and stearic fatty acids, pimaric-, isopimaric-, dehydroabietic-, abietic resin acids. *A* univariate analysis was used to evaluate the differences between the groups of variables, using the IBM SPSS Statistics 20 [34] software.

## Results and discussion

### Distribution of extractives

**Fig 3:**
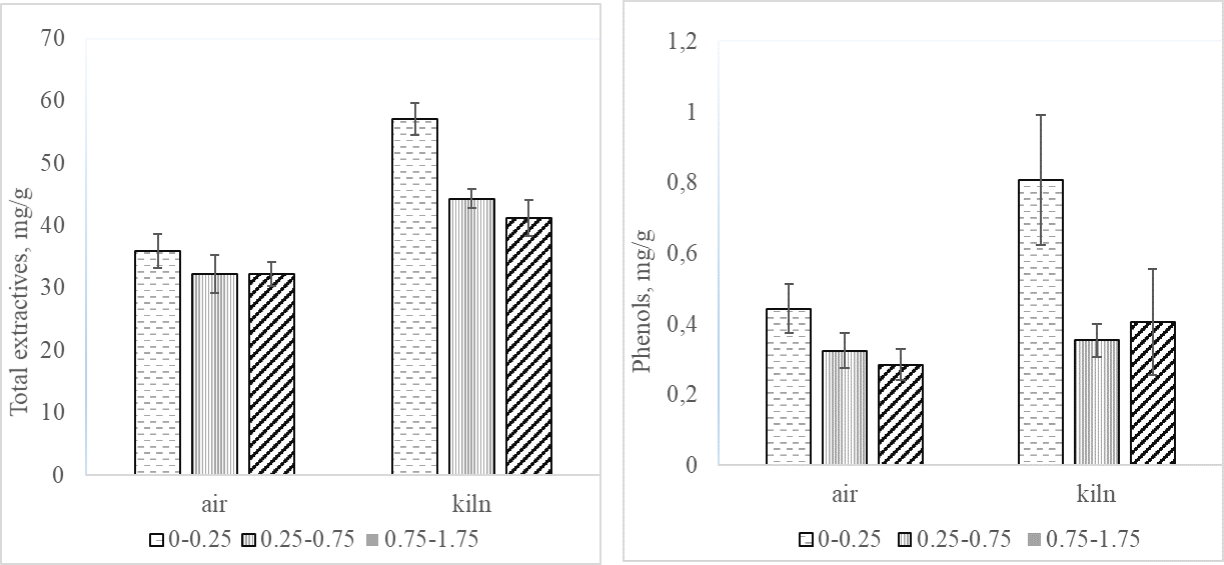
Total extractives content (A) and phenolic content (B) in air-dried and kiln-dried Scots pine sapwood boards.

The total extractives content in the three regions beneath the sapwood surface is shown in Fig 3A and 3B. The extractives content in the surface layers (i.e. 0–0.25 mm depth) of the boards was at the same level (32.2–56.6 mg g^-1^) as that reported by Dorado and Normark for the acetone extract from Scots pine sapwood [35,36,37]. The kiln-dried specimens showed a greater amount of total extractives in the surface layer than the air-dried samples, and the extractives content was lower in the two deepest layers (0.25–1.75 mm) than in the region closest to the surface (Fig 3).

The presence of a water evaporation front in capillaries near the wood surface during the drying process has been deduced from observations such as the enrichment of nutrients and reaction products some cell rows below the wood surface [6–9]. This can be explained by the movement of water-soluble nutrients together with the capillary flow of water during the early phases of wood drying and that they or their reaction products remain after evaporation of the capillary water. It is also possible that the observed enrichment of extractives in the uppermost layer is due to the migration of extractives during the capillary-transport phase of the drying, especially under kiln-drying conditions when the temperature in the wood is higher and the solubility and volatility of lipophilic substances is also higher [27,38]. It could not, however, be excluded that degradation of extractives takes place near the wood surface during drying or that the migration rate is related to the properties of migrating compounds. These issues are further discussed below.

Phenols in Scots pine are mainly found in the heartwood but also to some extent in the sapwood [22]. The phenolic content in the surface layers (i.e. 0–0.25 mm depth) seemed to be higher after kiln drying than after air drying at room temperature (Fig 3B). The scatter of the data was fairly large, but lower contents of phenols were observed in the lower surface layers. The significance, expressed as p-value, of the difference in the content of extractive materials between the layers was assessed by a F-test (Table 3). Low p-values (≤0.05) were found for extractives and phenols for both drying types. This suggests a clear difference in total extractives and phenolic content at different planing depths for both drying types and that also phenols migrate during both air-and kiln-drying of Scots pine. Weak boundary layer could be formed by enrichment of extractives on wood surfaces and hinder efficient adhesion with especially hydrophilic treatments. It is well known that linseed oil could be used to increase water repellency of wood but needs addition of biocides or active phenolic compounds to hinder mould growth when exposed under exterior conditions.

**Table 3:** The p-value for the significance of difference at different planing depths between air- and kiln-dried specimens.

Parameter	Air-dried	Kiln-dried
***Total extractives***	*0*.001*	*0*.001*
***Total phenols***	*0*.044*	*0*.022*
***Glycerol***	*0*.*280*	*0*.*481*
***Palmitic acid***	*0*.*385*	*0*.*460*
***Oleic acid***	*0*.*554*	*0*.*319*
***Linoleic acid***	*0*.*454*	*0*.*212*
***Stearic acid***	*0*.036*	*0*.*183*
***Pimaric acid***	*0*.*239*	*0*.034*
***Isopimaric acid***	*0*.*348*	*0*.028*
***Dehydroabietic acid***	*0*.*374*	*0*.*169*
***Abietic acid***	*0*.*478*	*0*.*266*

* Significance of difference between planing depth by F-test, the level of significance is p≤0.05.

Thus, knowledge of extractives and their composition on wood surfaces as a function of wood and process conditions is of great importance when exposed to exterior conditions.

The higher FC determined phenolic content in upper layer of air-dried boards could be favoured by dissolution in products of lipophilic origin which will migrate to wood surface [2]. The chemical changes such as oxidation of unsaturated fatty acids and natural degradation of resins during air-drying of wood occurs mainly due to that water is evaporated [1]. The evaporation of water changes also the physiological conditions of the timber cell walls and lower metabolic activity with time. Although, the degradation at kiln-drying could be more severe due to high concentration of hot water inside the kiln, the higher content of lipophilic compounds (i.e. total extractives including FC determined phenols) in kiln- than in air-dried boards suggests that migration towards surface is favoured probably due to pressure gradients inside the wood in the drying chamber [2]. The correlation coefficients between total extractives and FC-determined phenols for air-dried and kiln-dried specimens were 0.7 and 0.5 respectively that supports our hypothesis that performance of drying process influence not only distribution of hydrophilic but hydrophobic (lipophilic) wood constituents as well.

According to Fig 3 we have higher extractive amounts and phenols in the external (outer part) than internal (inner part) of boards. This could be controlled by the volatility, reactivity and migration rate of the individual extractives or group of extractives. Sugars are non-volatile and as they are readily soluble in water and will degrade only slowly under these drying conditions they will follow the migration of warm water during the capillary phase of drying and will be left and accumulated on the outermost part of the wood. The process could be seen as emptying a bath tub and only when the capillary is broken and such transport is stopped, during final drying, a receding evaporating front will be observed that is not supplied with migrated sugars.

Triglycerides and other more lipophilic substances are hardly dissolved in the water and should not be influenced by this water capillary process. As they are the major extractive compounds, the total extractives will therefore be less influenced by this process and actually smaller difference between the two drying processes is also seen than for monosaccharides [7,8]. Migration aptitude of phenols could be higher than the more lipophilic ones especially if they have not too high molecular weight and exist in glucoside form (Fig 3).

**Fig 4:**
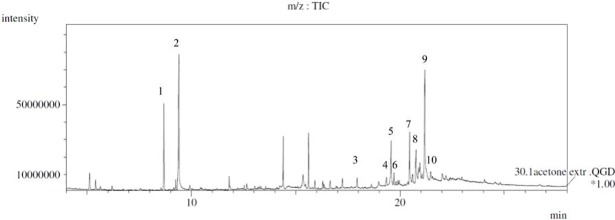
Chromatogram (GC-MS) of trimethylsilylated extractives from surface of kiln-dried board: 1. glycerol, 2. internal standard, 3. palmitic acid, 4. linoleic acid, 5. oleic acid, 6. stearic acid, 7. pimaric acid, 8. isopimaric acid, 9 dehydroabietic acid and 10. abietic acid.

Composition of isolated extractives from air- and kiln-dried board was studied using GC-MS as trimethylsilyl derivates (Fig 4).

For pimaric acid types, F-test analysis showed significant differences for kiln-dried boards and also for stearic acid in air-dried boards (Table 3). Oleic, palmitic, linoleic and stearic acids were found in the chromatograms (Fig 4), a finding in line with previous work; these are most common fatty acids in the sapwood of Scots pine [23]. The distribution of fatty acids within the surface regions was different from that of the other extractives and they were also found in larger amounts in the air-dried than in the kiln-dried boards, Table 4. On the other hand, more glycerol was found in the kiln-dried than in the air-dried specimens, indicating a more extensive degradation of triglycerides to glycerol and fatty acids under those conditions. The interpretation of the data is thus more complicated since fatty acids could be formed from the degradation of fats, but they could also be consumed in further oxidation reactions. The unsaturated linoleic acid has been reported as a major free fatty acid and fatty acid in triglycerides in Scots pine [23,24]. However, the variation in the amount of linoleic acid was high in the present study, Table 3. This is partly because it is not fully separated from other compounds such as oleic acid in the chromatogram (Fig 4). Additional uncertainty in linoleic acid data could be due to its oxidation into products such as hexanoic acid and heptanal which were found in the dried boards [39]. It is possible that oxidative degradation was greater in kiln-dried boards.

**Table 4:** The mean, range, standard error (SE) and coefficient of variation (CV in %), Δ mean of glycerol and fatty acids in air-dried and kiln-dried samples at different depths from the surface, mg/g.

Depth (mm)	Air-dried			Kiln-dried			Δ mean %
	Mean mg/g	Range (SE) mg/g	(CV) %	Mean mg/g	Range (SE) mg/g	(CV) %	
***Glycerol***							
0–0.25	0.43	0.12…1.05 (0.08)	60	0.82	0…1.68 (0.17)	64	+91
0.25–0.75	0.42a	0.08…0.80 (0.08)	62	1.03	0.28…3.05 (0.28)	87	+145
0.75–1.75	0.64a	0.08…1.61 (0.15)	72	1.28a	0…2.54 (0.33)	82	+100
***Palmitic acid***							
0–0.25	0.45	0…2.13 (0.25)	177	0.24	0…1.25 (0.14)	181	-47
0.25–0.75	0.11	0…0.36 (0.04)	120	0.09	0…0.47 (0.05)	163	-18
0.75–1.75	0.28	0…1.34 (0.16)	177	0.12	0…0.35 (0.04)	118	-57
***Oleic acid***							
0–0.25	3.63	0.49…25.05 (2.39)	208	0.73a	0…1.77 (0.17)	75	-80
0.25–0.75	1.35	0.61…3.54 (0.03)	63	1.73	0…7.26 (0.68)	125	+28
0.75–1.75	3.72	0.30…17.40 (1.80)	153	1.30	0…4.28 (0.38)	92	-65
***Linoleic acid***							
0–0.25	0.68	0…6.23 (0.62)	287	0.20a	0…0.48 (0.05)	80	-71
0.25–0.75	0.17a	0…0.45 (0.05)	91	0.09a	0…0.23 (0.03)	100	-47
0.75–1.75	0.07	0…0.56 (0.05)	236	0.09	0…0.67 (0.07)	233	-29
***Stearic acid***							
0–0.25	0.29	0.09…0.90 (0.08)	88	0.33	0…1.25 (0.11)	103	+14
0.25–0.75	0.13a	0…0.27 (0.02)	62	0.23	0.01…1.17 (0.11)	148	+77
0.75–1.75	0.25	0…2.04 (0.20)	252	0.08	0…0.52 (0.05)	200	-68

The larger amounts of the resin acids, dehydroabietic, pimaric, isopimaric and abietic acids were found in the surface layers (0–0.25 mm) of both groups of boards, Table 5, which suggests that resin acids migrate during both drying methods. A somewhat higher content of resin acids was found in the kiln-dried than in the air-dried boards. The content of pimaric- and isopimaric acids was clearly lower than that of dehydroabietic acid and corresponds to previously reported data for stemwood of 82–84 year old Scots pine in northern Sweden [22].

**Table 5:** The mean, range, standard error (SE) and coefficient of variation (CV), Δ mean of resin acids in air- and kiln-dried samples at different depth from the surface, mg/g.

Depth (mm)	Air-dried			Kiln-dried			Δ mean %
	Mean mg/g	Range (SE) mg/g	CV %	Mean mg/g	Range (SE) mg/g	CV %	
***Pimaric acid***							
0–0.25	1.58	0.38…6.69 (0.59)	119	1.94a	0.11…3.77 (0.40)	65	+23
0.25–0.75	0.73a	0.23…1.23 (0.11)	50	0.99a	0.23…2.00 (0.19)	62	+36
0.75–1.75	0.82	0.14…2.13 (0.22)	83	0.91	0…2.58 (0.25)	86	+11
***Isopimaric asid***							
0–0.25	1.05	0.28…3.34 (0.30)	90	1.49	0.10…3.03 (0.35)	75	+42
0.25–0.75	0.55a	0.14…1.13 (0.09)	55	0.77a	0.16…1.93 (0.16)	67	+40
0.75–1.75	0.51a	0.04…1.12 (0.12)	74	0.59a	0…1.43 (0.13)	71	+16
***Dehydroabietic acid***							
0–0.25	4.37	0.78…17.61 (1.56)	113	4.55a	0.32…7.58 (0.77)	54	+4
0.25–0.75	2.00a	0.50…3.19 (0.31)	48	2.89a	0.57…6.96 (0.62)	67	+45
0.75–1.75	2.49a	0.16…6.45(0.58)	73	2.65	0…9.65 (0.84)	100	+6
***Abietic acid***							
0–0.25	0.81	0.25…4.12 (0.37)	146	0.85	0.33…2.25 (0.18)	68	+5
0.25–0.75	0.33a	0…0.75 (0.06)	60	0.77	0.09…1.96 (0.23)	93	+133
0.75–1.75	0.56	0…1.89 (0.22)	121	0.44	0…1.17 (0.13)	93	+21

a–normally distributed according to the Kolmogorov-Smirnov test with Lilliefors significance correction, p≥0.05.

The amount of abietic acid was found to be rather low compared to dehydroabietic acid, Table 5. This corresponds to what has been reported for Scots pine sapwood in Northern Finland [22].

The content and distribution of dehydroabietic acid was found to be fairly similar in air-dried and kiln-dried boards, Table 5. An indication of formation of the oxidized product 7-ketodehydroabietic acid was found in the mass peaks of 386, 371, 268 and 253, but the amounts were generally low or non-detectable in the investigated samples. This compound has been found in sawdust during storage in piles [40]. Under these conditions, microbiological oxidation of extractives can occur raising the temperature inside the pile.

## Conclusion

This study describes the distribution of lipophilic extractive chemicals after air-drying and kiln-drying of green Scots pine sapwood. Enrichment of total extractives, phenols and pimaric acids was observed in the surface layers (0–0.25 mm below the original surface) compared to regions some 1.5 mm below the surface. A similar tendency was observed for abietane types of resin acids during the drying operations. The extent of enrichment was more pronounced after drying at a higher temperature than after drying at room temperature. These data confirm our assumption that migration of hydrophobic extractives occurs especially during kiln drying at a moderate to high temperature. A higher mass ratio was found for phenols and resin acids than for total extractives when the surface and the regions beneath were compared. From the results we suggest that the evaporating front is related not only to conditions during drying but also to the migration ability of the extractives.

No clear indication of the presence of an evaporation front was found in the fatty acids distribution. The critical point of our study is the understanding of processes that occur below the surface. The fatty acids are also components of triglycerides and the amount of glycerol and the presence of degradation products such as hexanal and hexanoic acids provide evidence for the necessity of a more detailed and controlled experimental design in order to understand the complexity of the chemical processes occurring during natural and artificial drying. The development of robust predictive models for the assessment of wood properties requires more detailed studies of the chemistry of wood drying. That makes it possible to calibrate an on-site evaluation tool for the properties of dried timber and a better sorting for product development.

## Supporting information

S1 Table Data set of lipophilic extractives pine from air-dried and kiln-dried sapwood Scots pine. (DOCX)
