# Supplementary material for: Distribution of low-molecular lipophilic extractives beneath the surface of air- and kiln-dried Scots pine sapwood boards
Source: PLoS One. 2018 Oct 10;13(10):e0204212. doi: 10.1371/journal.pone.0204212 (PMC6179209; doi:10.1371/journal.pone.0204212)
Supplement: S1 Table — (DOCX) [file pone.0204212.s001.docx]

**S1 Table. Data set of lipophilic extractives pine from air-dried and kiln-dried sapwood Scots pine, mg/g. (DOC)**

| Board | Drying | Pl_Depth | Glycerol | Palmitic | Oleic | Linoleic | Stearic | Pimaric | Isopimaric | Dehydroabietic | Abietic | Extr | Phenols |
| --- | --- | --- | --- | --- | --- | --- | --- | --- | --- | --- | --- | --- | --- |
| 2 | air | 0…0.25 | 0,548066 | 1,771422 | 2,047531 | 0,150412 | 0,214922 | 1,315608 | 0,708227 | 3,477917 | 0,760895 | 47,08146 | 0,38136 |
| 2 | air | 0.25…0.75 | 0,372917 | 0,000000 | 1,56395 | 0,180173 | 0,167325 | 0,721438 | 0,376829 | 2,313299 | 0,361006 | 40,37833 | 0,375518 |
| 2 | air | 0.75…1.75 | 0,408828 | 0,111367 | 1,164815 | 0,071987 | 0,025439 | 0,450653 | 0,237083 | 1,55532 | 0,15884 | 35,99329 | 0,179966 |
| 5 | air | 0…0.25 | 0,495282 | 2,13298 | 25,05234 | 6,225504 | 0,903579 | 6,687396 | 3,342869 | 17,61476 | 4,119094 | 45,17893 | 0,813221 |
| 5 | air | 0.25…0.75 | 0,713598 | 0,205132 | 3,539936 | 0,418516 | 0,265887 | 1,162636 | 0,796203 | 2,686989 | 0,749426 | 38,31492 | 0,490431 |
| 5 | air | 0.75…1.75 | 0,478501 | 0,000000 | 0,303341 | 0,000000 | 0,000000 | 0,139871 | 0,038006 | 0,162592 | 0,14912 | 37,52754 | 0,55916 |
| 7 | air | 0…0.25 | 0,241808 | 0,000000 | 1,094776 | 0,035599 | 0,086986 | 0,381058 | 0,282911 | 1,402526 | 0,306999 | 30,74944 | 0,282895 |
| 7 | air | 0.25…0.75 | 0,079643 | 0,000000 | 0,649385 | 0,01068 | 0,065007 | 0,23197 | 0,143512 | 1,008293 | 0,000000 | 31,7332 | 0,231652 |
| 7 | air | 0.75…1.75 | 0,079148 | 0,000000 | 0,73662 | 0,000000 | 0,000000 | 0,293702 | 0,229484 | 1,578129 | 0,000000 | 30,16828 | 0,187043 |
| 12 | air | 0…0.25 | 0,123983 | 0,287758 | 1,312483 | 0,000000 | 0,530558 | 0,556874 | 0,559131 | 2,039398 | 0,272337 | 30,12418 | 0,237981 |
| 12 | air | 0.25…0.75 | 0,108742 | 0,358811 | 0,61247 | 0,028718 | 0,000000 | 0,433107 | 0,323844 | 1,337162 | 0,243112 | 31,06835 | 0,192624 |
| 12 | air | 0.75…1.75 | 0,224975 | 0,000000 | 0,917398 | 0,009698 | 0,000000 | 0,423526 | 0,305708 | 1,476253 | 0,137537 | 26,95224 | 0,164409 |
| 14 | air | 0…0.25 | 0,369698 | 0,099897 | 1,063596 | 0,053142 | 0,216843 | 1,663397 | 1,576286 | 3,929874 | 0,349899 | 49,06973 | 0,628093 |
| 14 | air | 0.25…0.75 | 0,454551 | 0,000000 | 1,019275 | 0,000000 | 0,062006 | 1,18656 | 1,129441 | 3,044781 | 0,347247 | 43,14301 | 0,586745 |
| 14 | air | 0.75…1.75 | 0,805314 | 0,000000 | 1,113701 | 0,036738 | 0,124067 | 1,085182 | 1,120125 | 3,51862 | 0,436719 | 41,35579 | 0,479727 |
| 20 | air | 0…0.25 | 0,38875 | 0,000000 | 1,070705 | 0,077291 | 0,146194 | 0,781811 | 0,744615 | 2,519092 | 0,470439 | 25,16447 | 0,221447 |
| 20 | air | 0.25…0.75 | 0,33487 | 0,115074 | 1,621816 | 0,14254 | 0,187662 | 0,691127 | 0,749245 | 3,006209 | 0,424687 | 27,0935 | 0,281772 |
| 20 | air | 0.75…1.75 | 0,797473 | 0,108644 | 1,698723 | 0,000000 | 0,145543 | 0,515594 | 0,469872 | 2,710944 | 0,409678 | 24,25715 | 0,184354 |
| 27 | air | 0…0.25 | 0,36977 | 0,000000 | 0,857878 | 0,118038 | 0,140776 | 0,752565 | 0,512698 | 2,576344 | 0,246364 | 33,43844 | 0,397917 |
| 27 | air | 0.25…0.75 | 0,516759 | 0,165738 | 0,904783 | 0,088707 | 0,110961 | 0,622153 | 0,373007 | 1,740333 | 0,308225 | 38,32647 | 0,329608 |
| 27 | air | 0.75…1.75 | 0,997389 | 1,345602 | 17,40355 | 0,000000 | 0,000000 | 2,128836 | 0,816573 | 3,894027 | 1,740405 | 40,0003 | 0,268002 |
| 28 | air | 0…0.25 | 0,217589 | 0,227733 | 2,458674 | 0,065792 | 0,399668 | 2,153737 | 1,67307 | 6,574356 | 0,941347 | 28,89907 | 0,378578 |
| 28 | air | 0.25…0.75 | 0,136135 | 0,000000 | 0,895063 | 0,177503 | 0,094294 | 0,306853 | 0,294759 | 1,193925 | 0,118621 | 30,18229 | 0,211276 |
| 28 | air | 0.75…1.75 | 0,210339 | 0,000000 | 0,929281 | 0,000000 | 0,000000 | 0,27502 | 0,143016 | 0,786494 | 0,170737 | 27,65008 | 0,188021 |
| 30 | air | 0…0.25 | 1,054243 | 0,000000 | 0,895022 | 0,045037 | 0,100082 | 0,852797 | 0,781615 | 2,756344 | 0,353341 | 39,19885 | 0,791817 |
| 30 | air | 0.25…0.75 | 0,671287 | 0,221007 | 1,503308 | 0,449625 | 0,174597 | 0,701018 | 0,596912 | 0,50217 | 0,349657 | 8,240547 | 0,084054 |
| 30 | air | 0.75…1.75 | 0,777756 | 1,065888 | 10,66327 | 0,060221 | 2,036461 | 1,756008 | 0,906122 | 6,446402 | 1,887389 | 28,48342 | 0,264896 |
| 33 | air | 0…0.25 | 0,512777 | 0,000000 | 0,488388 | 0,000000 | 0,177422 | 0,613128 | 0,283089 | 0,778021 | 0,313168 | 30,53454 | 0,305345 |
| 33 | air | 0.25…0.75 | 0,795368 | 0,000000 | 1,176945 | 0,239952 | 0,165314 | 1,229228 | 0,674474 | 3,192844 | 0,368417 | 33,7207 | 0,461974 |
| 33 | air | 0.75…1.75 | 1,613153 | 0,167555 | 2,309482 | 0,555877 | 0,210871 | 1,179423 | 0,799475 | 2,795068 | 0,545945 | 30,28523 | 0,375537 |
| 2C | kiln | 0…0.25 | 0,744775 | 1,254856 | 0,000000 | 0,36268 | 0,320281 | 2,476036 | 1,169689 | 5,93856 | 1,194276 | 53,20583 | 0,207503 |
| 2С | kiln | 0.25…0.75 | 0,513345 | 0,118738 | 0,466252 | 0,118754 | 0,090153 | 0,82421 | 0,297195 | 2,235569 | 0,333073 | 40,82805 | 0,236803 |
| 2С | kiln | 0.75…1.75 | 0,602586 | 0,254636 | 0,841523 | 0,000000 | 0,000000 | 0,594816 | 0,281592 | 1,631302 | 0,347615 | 34,33341 | 0,0515 |
| 5D | kiln | 0…0.25 | 0,000000 | 0,000000 | 0,23886 | 0,000000 | 0,000000 | 0,111469 | 0,102333 | 0,324956 | 0,479792 | 63,88312 | 0,006388 |
| 5D | kiln | 0.25…0.75 | 1,623769 | 0,000000 | 3,000499 | 0,226177 | 0,182172 | 1,537705 | 1,174608 | 4,411384 | 0,548442 | 48,82549 | 0,45896 |
| 5D | kiln | 0.75…1.75 | 2,372335 | 0,348685 | 1,223574 | 0,000000 | 0,000000 | 0,418171 | 0,254794 | 0,754465 | 0,117857 | 43,72797 | 0,183657 |
| 7B | kiln | 0…0.25 | 0,661742 | 0,064058 | 0,743683 | 0,215289 | 0,196227 | 0,829756 | 0,645342 | 2,29758 | 0,331476 | 41,86167 | 0,506526 |
| 7B | kiln | 0.25…0.75 | 0,284267 | 0,000000 | 0,221148 | 0,000000 | 0,18342 | 0,228445 | 0,160695 | 0,758167 | 0,087757 | 37,92759 | 0,128954 |
| 7B | kiln | 0.75…1.75 | 0,702075 | 0,000000 | 0,546185 | 0,000000 | 0,143988 | 0,564207 | 0,396879 | 1,872498 | 0,216738 | 35,894 | 0,107682 |
| 12A | kiln | 0…0.25 | 0,551509 | 0,000000 | 1,14917 | 0,484073 | 0,350642 | 3,770454 | 2,827425 | 7,088144 | 0,461246 | 56,68202 | 0,680184 |
| 12A | kiln | 0.25…0.75 | 0,483721 | 0,126728 | 7,257939 | 0,005333 | 1,168377 | 1,998943 | 0,888026 | 6,963731 | 1,413741 | 42,748 | 0,461678 |
| 12A | kiln | 0.75…1.75 | 0,000000 | 0,000000 | 1,309829 | 0,665165 | 0,517351 | 2,583703 | 0,91084 | 3,575584 | 1,093432 | 61,79466 | 1,062868 |
| 14C | kiln | 0…0.25 | 0,666162 | 0,292826 | 1,77299 | 0,072366 | 1,249394 | 3,350036 | 3,130593 | 7,5274 | 1,312266 | 64,32523 | 1,222179 |
| 14C | kiln | 0.25…0.75 | 0,42602 | 0,000000 | 0,721004 | 0,000000 | 0,012079 | 0,715684 | 0,713837 | 2,122479 | 0,176817 | 50,51033 | 0,303062 |
| 14C | kiln | 0.75…1.75 | 0,35435 | 0,000000 | 0,796446 | 0,000000 | 0,000000 | 0,757414 | 0,707015 | 2,16379 | 0,100375 | 49,08886 | 0,279807 |
| 20A | kiln | 0…0.25 | 0,569109 | 0,000000 | 0,000000 | 0,2625 | 0,170833 | 0,947232 | 0,734761 | 3,396406 | 0,543411 | 54,96032 | 0,835397 |
| 20A | kiln | 0.25…0.75 | 0,380307 | 0,000000 | 0,950207 | 0,109016 | 0,160656 | 0,629499 | 0,450733 | 2,581105 | 0,261174 | 36,64074 | 0,329767 |
| 20A | kiln | 0.75…1.75 | 0,000000 | 0,000000 | 0,000000 | 0,000000 | 0,000000 | 0,000000 | 0,000000 | 0,000000 | 0,000000 | 40,83046 | 0,273564 |
| 27B | kiln | 0…0.25 | 1,186688 | 0,806987 | 0,947321 | 0,000000 | 0,2592 | 1,786722 | 1,325837 | 4,368872 | 0,736825 | 48,00000 | 0,508800 |
| 27B | kiln | 0.25…0.75 | 1,243544 | 0,000000 | 0,000000 | 0,000000 | 0,097102 | 0,409151 | 0,827171 | 0,572198 | 1,963832 | 49,43369 | 0,400413 |
| 27B | kiln | 0.75…1.75 | 2,453157 | 0,112571 | 1,356137 | 0,000000 | 0,000000 | 1,188282 | 0,91479 | 3,080785 | 0,420103 | 44,17726 | 0,238557 |
| 28A | kiln | 0…0.25 | 0,484696 | 0,000000 | 0,747882 | 0,090361 | 0,164068 | 1,34521 | 0,962967 | 4,139399 | 0,617761 | 59,62389 | 1,025531 |
| 28A | kiln | 0.25…0.75 | 0,51932 | 0,465597 | 2,49867 | 0,036438 | 0,086463 | 1,360912 | 0,902825 | 3,33666 | 1,846029 | 43,64313 | 0,187665 |
| 28A | kiln | 0.75…1.75 | 2,536891 | 0,30171 | 4,280318 | 0,000000 | 0,000000 | 1,92218 | 1,426766 | 9,650866 | 1,169683 | 31,82479 | 0,136847 |
| 30C | kiln | 0…0.25 | 1,609099 | 0,000000 | 1,000296 | 0,302046 | 0,314886 | 3,520594 | 3,132764 | 7,584099 | 2,245718 | 69,70297 | 2,063208 |
| 30C | kiln | 0.25…0.75 | 3,045841 | 0,000000 | 1,29848 | 0,221324 | 0,227148 | 1,69414 | 1,931793 | 4,373002 | 0,865555 | 45,98142 | 0,427627 |
| 30C | kiln | 0.75…1.75 | 1,723957 | 0,172567 | 0,562009 | 0,000000 | 0,000000 | 0,536801 | 0,588545 | 1,850413 | 0,697097 | 38,77216 | 0,224879 |
| 33D | kiln | 0…0.25 | 1,67847 | 0,000000 | 0,688398 | 0,218614 | 0,231929 | 1,289887 | 0,894406 | 2,82696 | 0,620994 | 58,42098 | 1,028209 |
| 33D | kiln | 0.25…0.75 | 1,798026 | 0,22768 | 0,925948 | 0,151081 | 0,06511 | 0,488389 | 0,357926 | 1,55386 | 0,225332 | 46,35668 | 0,602637 |
| 33D | kiln | 0.75…1.75 | 2,073579 | 0,000000 | 2,083121 | 0,200078 | 0,127432 | 0,516553 | 0,399774 | 1,930896 | 0,26444 | 32,28687 | 1,498235 |
